# Important Correction

**Published:** 1852-12

**Authors:** 


*Important Correction.*— We find the following in the New York Med. Gazette: "In Christison's Dispensatory, edited by Griffith, the dose of hydrargyri biniodidum is, through mistake, set down at from *one to four grains*, whereas it should be from *one-sixteenth to one-fourth* of a grain. Those who have the work should be careful to correct this, as a mistake of the kind might prove fatal."

